# Measurement of Spray Drift with a Specifically Designed Lidar System

**DOI:** 10.3390/s16040499

**Published:** 2016-04-08

**Authors:** Eduard Gregorio, Xavier Torrent, Santiago Planas de Martí, Francesc Solanelles, Ricardo Sanz, Francesc Rocadenbosch, Joan Masip, Manel Ribes-Dasi, Joan R. Rosell-Polo

**Affiliations:** 1 Research Group in AgroICT & Precision Agriculture, Department of Agricultural and Forest Engineering, Universitat de Lleida (UdL)—Agrotecnio Center, Edifici CREA, c/Pere de Cabrera s/n, Lleida 25001, Spain; xavier.torrent@eagrof.udl.cat (X.T.); splanas@eagrof.udl.cat (S.P.d.M.); rsanz@eagrof.udl.cat (R.S.); jmv@eagrof.udl.cat (J.M.); manelo@eagrof.udl.cat (M.R.-D.); jr.rosell@eagrof.udl.cat (J.R.R.-P.); 2 Department of Agriculture, Livestock, Fisheries and Food, Generalitat de Catalunya, Lleida 25198, Spain; fsolanelles@gencat.cat; 3 Remote Sensing Laboratory, Department of Signal Theory and Communications, Universitat Politècnica de Catalunya (UPC)/IEEC, Barcelona 08034, Spain; roca@tsc.upc.edu

**Keywords:** lidar, spray drift, pesticide, laser, remote sensing, agriculture

## Abstract

Field measurements of spray drift are usually carried out by passive collectors and tracers. However, these methods are labour- and time-intensive and only provide point- and time-integrated measurements. Unlike these methods, the light detection and ranging (lidar) technique allows real-time measurements, obtaining information with temporal and spatial resolution. Recently, the authors have developed the first eye-safe lidar system specifically designed for spray drift monitoring. This prototype is based on a 1534 nm erbium-doped glass laser and an 80 mm diameter telescope, has scanning capability, and is easily transportable. This paper presents the results of the first experimental campaign carried out with this instrument. High coefficients of determination (R^2^ > 0.85) were observed by comparing lidar measurements of the spray drift with those obtained by horizontal collectors. Furthermore, the lidar system allowed an assessment of the drift reduction potential (DRP) when comparing low-drift nozzles with standard ones, resulting in a DRP of 57% (preliminary result) for the tested nozzles. The lidar system was also used for monitoring the evolution of the spray flux over the canopy and to generate 2-D images of these plumes. The developed instrument is an advantageous alternative to passive collectors and opens the possibility of new methods for field measurement of spray drift.

## 1. Introduction

The ISO 22866 standard [1] defines spray drift in agricultural pesticide applications as the quantity of plant protection product that is carried out of the sprayer (treated) area by the action of air currents during the application process. The spray drift, which is usually measured near to where the applications are carried out, is made up of droplets, although it can also be composed of solid particles or vapour. These last two fractions can travel long distances in the atmosphere till they are deposited far away from the source [2,3]. Spray droplets that are more likely to drift away are those with a diameter of less than 100 µm [4].

Spray drift is a serious threat to the environment, as it can contaminate surface water bodies [5], bystanders [6], and nearby urban [7] or natural areas. To prevent the contamination of water, risk mitigation schemes have been developed. For instance, in Germany they were defined according to the results of spray drift measurements in different crops [8]. In relation to the spray application technology, several spray drift reduction techniques, such as air-injection nozzles and tunnel sprayers, have been introduced to decrease the contamination hazard [9].

Spray drift can be measured in two different ways near the treated area. The use of one or another will depend on the aim of the measurement. In order to determine the spray drift that can reach the surface water, the spray drift deposited on the ground at different distances from the treated area is measured. On the other hand, the measurement of the airborne spray drift, up to a given height, can be more useful for the assessment of risk to bystanders. Passive collectors are the most common measurement techniques, both for ground and airborne spray drift [10]. However, there are several drawbacks to their use [11,12], including the fact that it is a time-consuming, single-point, time-averaged sampling methodology with calculation inaccuracies because of the difficulty of knowing the spray collection efficiency [13]. Moreover, measurements with passive collectors are only possible if chemical tracers are used in the spray application [14,15].

The difficulties of using passive collectors for the measurement of spray drift have prompted the development of alternative methodologies that can overcome some of its disadvantages. Among these, measurements carried out in wind tunnels [16,17] or by using test benches [18,19] can be cited. Moreover, a drift assessment of the nozzles can be obtained from the measurement of their droplet size [20,21]. Unlike field tests, these methodologies allow drift measurements to be taken in controlled and repeatable conditions. Despite their advantages, these indirect methods cannot reproduce all real application conditions and complete drift studies must be conducted in the field.

The light detection and ranging (lidar) technique is one of the most promising alternatives for field measurement of spray drift. Compared to passive collectors, lidar systems consume less labour and time resources, provide measurements with high temporal and spatial resolution, and no subsequent chemical analyses are required. In previous works, lidar systems have been used to study the spray plume generated in aerial applications [22,23,24], to validate theoretical spray-transport models [25], to assess the influence of atmospheric stability over spray drift movement and dispersal [26,27], and to quantify the concentration of the drift cloud [11,28,29,30]. Most of the lidar systems used in previous works are complex, expensive, present an optomechanical configuration not suited for near-field measurements and usually they are not eye-safe. Due to these factors, lidar systems have been used only in a limited way in spray drift studies.

Recently, the authors developed the first lidar system specifically designed for spray drift monitoring [31]. It is a low-cost, eye-safe, easily transportable system, with scanning capability. This paper presents the first experimental field campaign conducted with the developed prototype. The main objective of this experimental work was to test the lidar system in real conditions, studying its potential application instead of conventional passive collectors. The lidar system was used for monitoring the spray flux over the canopy and its temporal evolution, which are indicators of the drift potential. Furthermore, lidar measurements of the spray were compared to those obtained using passive collectors and following the ISO 22866 standard [1]. In all of the experiments, both standard and low-drift nozzles were tested.

## 2. Materials and Methods

### 2.1. Study Site

The spray tests were carried out between 11 and 21 November 2014, at a field owned by the Institut de Recerca i Tecnologia Agroalimentàries (IRTA) in Gimenells (lat. 41**°**39′11′′N, long. 0**°**23′28′′E, elev. 259 m) located 25 km from Lleida, Spain. In order to follow the ISO 22866 standard [1], an experimental intensive apple orchard (grow stage, BBCH: 92) adjacent to an uncultivated area downstream of the prevailing winds (west-east direction) was chosen. The orchard consists of a germplasm bank of apple trees and their crossbreeds, with a development similar to a commercial variety. The trees were planted in 2011 on rows with a spacing of 3.45 × 0.60 m (between rows × between trees). Tree dimensions at the time of trials were 2.80 × 1.90 m (average height × average depth).

One air-assisted sprayer (Teyme Eolo 2091, Teyme Tecnología Agrícola SL, Torre-Serona, Spain) with five operating nozzles on each side (left/right) was used. Two nozzle types were tested: (1) standard hollow cone (Albuz ATR 80 Grey, Saint-Gobain, Evreux, France) and (2) air-injected low-drift (Albuz TVI 80 03, Blue). These two nozzle types were selected because at a working pressure of 1 MPa, they produce similar flow rates (2.09 and 2.24 L·min^−1^ for the ATR and for the TVI, respectively).

Table 1 presents the operating conditions of all the tests. For each test, the date the test was conducted, start time, duration of the spraying, lidar operational mode, passive collectors that were used, sprayer operational mode, and nozzle model employed are shown.

### 2.2. Lidar System

**Figure 1:**
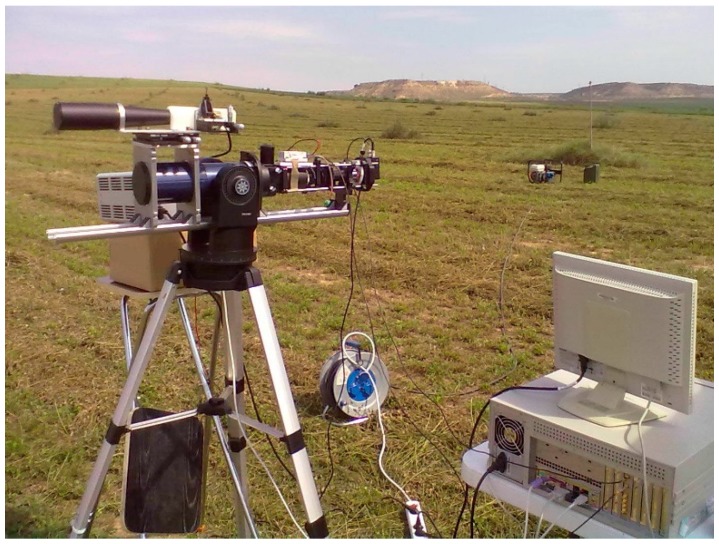
Picture of the developed lidar system deployed in the field.

The developed lidar system [31] is based on a 1534 nm 3 mJ pulse-energy erbium-doped glass laser. The eye safety [32] is achieved by using a 20x beam expander. An XY miniature translation stage and a pitch & yaw platform are used to adjust the position and tilting of the laser emitter relative to the beam expander. Likewise, a high-load pitch & yaw platform is used to adjust the tilt angle between the emission and receiving optical axes. The backscattered signal is captured by an 80 mm diameter telescope. Through several optical elements (camera lens, beam reducer, interference filter, and microscope lens), the light collected by the telescope is focused on the photosensitive surface of an InGaAs avalanche photodiode module. An analogue-digital converter (ADC) digitizes the analogue signal from the photodetector and transmits it to a computer (Figure 1). Lidar system specifications are listed in Table 2.

As shown in Figure 1, the emission and receiving systems are held by a telescope mount attached to a tripod. This mount allows scanning in both azimuth and elevation with adjustable speed between 0.008 and 4 degrees/s.

### 2.3. Meteorological Measurements

A portable weather station equipped with a temperature (model MCP9808, Adafruit Industriels LLC, New York, NY, USA), humidity (model HIH 5030/5031, Honeywell, Golden Valley, MN, USA), wind speed (model Watson 8681-WSS, W & S, Hockley, Essex, UK), and wind direction (based on ACE-128 encoder, Bourns, Riverside, CA, USA) sensor was used.

All sensors were positioned at a height of 7 m and the temperature and humidity sensors were also positioned at 4 m. Wind direction was assumed to be 0**°** when the wind approached the drift measurement area (to the foreground in Figure 3) in a direction orthogonal to the rows. Positive wind direction values mean a deviation to the left in the same figure. All meteorological measurements were taken at a frequency of 1 Hz. Micrometeorological conditions during the tests are shown in Table 3. Average values were calculated taking into account data acquired during the spraying duration.

Meteorological data presented in Table 3 meets acceptable conditions for field measurement of spray drift according to ISO 22866 standard [1]. Thus, in all trials, the wind speed was close to or greater than 1 m/s, wind direction was at 90**°** ± 30**°** to the downwind edge of the directly sprayed area (during the period of spraying) and temperatures were maintained within the 5–35 **°**C range.

### 2.4. Lidar Measurement Configurations

**Figure 2:**
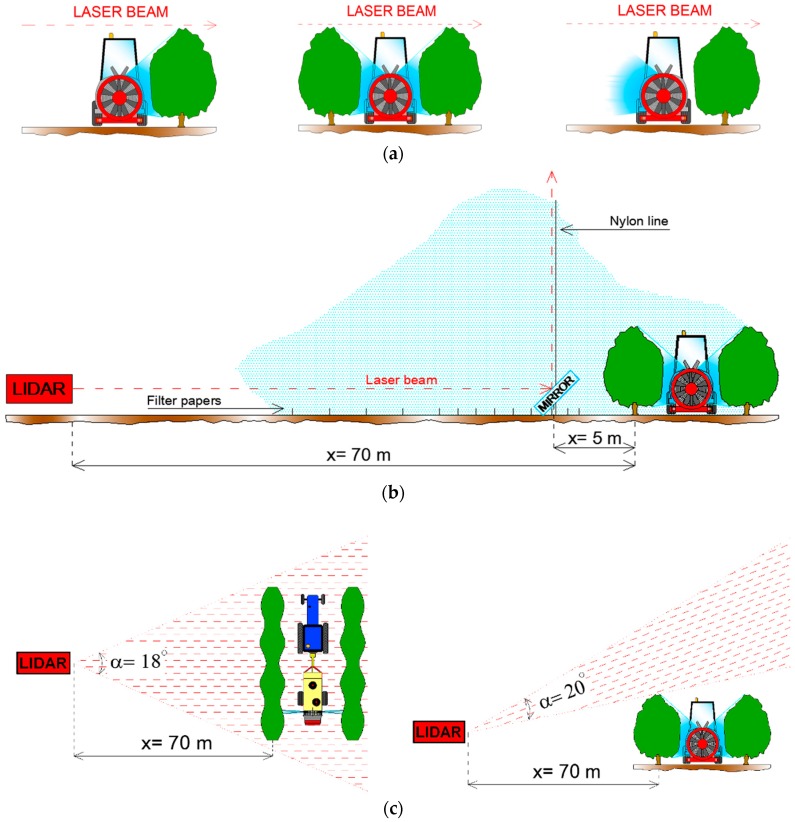
(**a**) Lidar measurements of the spray flux over the canopy (staring mode). Spraying sequence followed by the sprayer; (**b**) Comparison between lidar and passive collector (ISO 22866 [1]) measurements of the spray drift; (**c**) Position of the lidar system and air-assisted sprayer while the lidar was scanning the spray plume: (left) PPI scanning configuration; (right) RHI (vertical) scanning configuration.

Figure 2 illustrates the three experimental test setup considered. The first experiment (tests T1 and T2) was aimed at measuring the spray flux over the canopy. As shown in Figure 2a, the lidar system was kept on a static position (staring mode) while performing spray flux measurements over the trees. In the second experiment (tests T3–T6), spray drift measurements carried out with the lidar were compared against those from passive collectors following the ISO 22866 standard [1]. In this experiment, a mirror (Figure 2b) was used to vertically deflect the laser beam. This setup also allows simultaneous comparison between the spray drift measured by the lidar and both the horizontal collectors (filter papers along the horizontal path) and the vertical collectors (nylon lines along the vertical path). In the third experiment (tests T7–T10), the lidar scanned the spray plume over the canopy. Figure 2c shows the two scanning strategies followed.

### 2.5. Spray Flux Measurement over the Crop Canopy

In tests T1 and T2 the lidar system was placed at a distance of 70 m from the first tree row, pointing statically (staring mode) above them and emitting at a pulse repetition rate of 1 Hz. This distance ensures that the first meaningful lidar backscatter sample is well above the range of full overlap [33], which is about 30 m in this lidar sensor, and considering a sampling distance of 40 m from the first tree row. In both tests, the application sequence shown in Figure 2a was followed: (1) the sprayer moved along the external alley spraying the first tree row using only the nozzles on the right side; (2) the sprayer moved along the first internal alley spraying the first and second tree rows using the nozzles of both sides; and (3) the sprayer moved along the external alley, but in this case, the application was carried out with only the nozzles on the left side.

Lidar signal can be represented by means of different colours through range-time intensity plots (RTI). To obtain these RTI plots, the background signal from time-averaged measurements carried out before the start of the spraying was firstly calculated. To obtain calibrated measurements, this background signal was subtracted from the original measurements (raw data). Since the backscattered lidar signal depends on the inverse square of the distance [33,34], calibrated measurements were range-corrected to allow their comparison.

### 2.6. Comparison between Spray Drift Measurements Using the Lidar and the ISO 22866 Methodology

Four spray tests (T3–T6) were carried out to compare the measurements performed with the lidar system and the results obtained from the passive collectors following the ISO 22866 standard [35]. In this experiment, the spray liquid was an aqueous solution (1 g·L^−1^) of brilliant sulfoflavine (BSF, Biovalley, Marne La Vallée, France), while in the other tests, tap water was used. For the measurement of ground drift, filter papers (515 × 65 mm) were placed along three lines 1.5 m downwind from the first tree row, every 2.5 m from 2.5–20 m, and every 5 m from 20–40 m. For the measurement of airborne drift, two 6 m height, 2 mm diameter nylon lines were placed on each pole at 5 and 10 m downwind. Also, water-sensitive paper sheets (26 × 76 mm) were attached to the vertical pole at 5 m downwind and separated by 0.5 m from each other.

**Figure 3:**
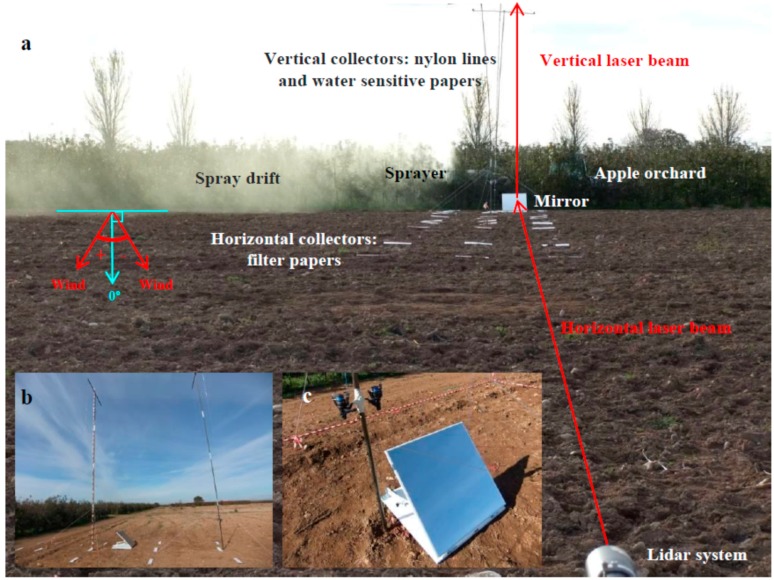
Experimental setup of the ISO 22866 [1] and simultaneous lidar measurements: (**a**) General view of the apple orchard experimental site with relative position of the sprayer, lidar system, mirror, and passive collectors; (**b**) Side-view detail showing the two poles, each one holding two nylon lines; (**c**) Magnified detail of the mirror placed near the pole at 5 m downwind from the first tree row.

The lidar system was placed at a distance of 70 m from the trees working in staring mode as in previous tests and pointing to an 80 × 80 cm square mirror (Mirox 3G, AGC Glass Europe, Louvain-la-Neuve, Belgium) with a thickness of 5 mm and 86% of light reflection. The mirror (45**°** inclination) was placed near the pole, at 5 m from the first row of trees (Figure 2b). The laser beam (1 Hz repetition rate) was emitted horizontally with a path parallel to the horizontal collectors. The mirror deflected the beam vertically, so its path was parallel to vertical collectors. This experimental setup allowed a simultaneous comparison of lidar measurements with both horizontal and vertical collectors. Figure 3 shows a picture of the experimental site along with the location of the machinery and measuring systems.

Determination of BSF concentration in nylon lines and filter papers was carried out with a fluorescence spectrophotometer (LS 30 Luminescence Spectrometer, Perkin Elmer, Waltham, MA, USA) following the methodology described by Gregorio *et al.* [11]. Water-sensitive papers were processed using specific software (ImageJ, version 1.49c, National Institute of Health, Bethesda, MD, USA). The images were taken with 24 pixels·mm^−1^ resolution. In each image, the coverage (percentage of surface covered by all the droplets present in the image) and the impact numbers were obtained.

Time-integrated lidar signal (with spatial resolution) was calculated for comparison with passive collector data. This signal is calculated by adding together the range-corrected background-subtracted lidar data throughout all the measurement period.

Drift reduction potential (DRP) is computed from both passive collector and lidar measurements. DRP is the drift percentage reduction in the studied spraying relative to a reference spraying [17]. To make this calculation from passive collector measurements, numerical integration of the area under the fallout deposit curves is carried out [21,36]. Similarly, the DRP can also be computed from time-integrated lidar signal curves.

### 2.7. Lidar Scanning of the Spray Plume over the Crop Canopy

Lidar scanning of the spray plume over the vegetation was carried out following two strategies [37]: plan position indicator (PPI) scans and range-height indicator (RHI) scans. PPI scans (tests T7 and T9) are based on maintaining a constant elevation angle and modifying the azimuth. In the PPI scans, the laser beam was emitted with a small elevation angle (<8°) in order to measure above the trees. This elevation angle was experimentally adjusted to avoid laser impacts on tree branches. RHI scans (tests T8 and T10), also known as vertical scans, are based on maintaining a constant azimuth angle and changing the elevation. The lidar system was located at a distance of 70 m from the first tree row, while the sprayer was placed at the first internal alley and kept it in static position throughout all the tests (Figure 2c). The following sequence was undertaken during these tests: (1) the lidar system scanned following a counterclockwise (PPI scans) or an upward (RHI scans) movement for 5 s with the laser emitting at a repetition rate of 5 Hz; (2) movement in the opposite direction for 5 s with no laser emission until returning to the initial position; (3) waiting for 5 s with the lidar in static position without emission. As shown in Figure 2c, PPI scans covered an angular range of 18° with a resolution of 0.72°, while RHI scans (with a slightly higher speed) covered 20**°** with a resolution of 0.8°. With the proposed scanning time duration and angular ranges, the lidar system was able to sweep 22 m width (PPI scan) and 24 m height (RHI scan) over the canopy. These values were enough to cover the entire cloud drift. Each of the previous cycles was completed in 15 s, preforming a total of 4 to 6 consecutive cycles per test. Spraying followed the next sequence: cycle 1, no application was made and lidar measurements were used to obtain the background signal; cycle 2, application was conducted for 15 s using the nozzles of both sides; cycle 3 and subsequent cycles, no application was made and the lidar system monitored the spray plume remaining over the crop.

Lidar signal processing was carried out in a similar way as in the previous tests (background subtraction and range correction) except that in this case, two coordinates within a plane (PPI plane or RHI plane) are assigned to each measurement. The coordinates of each measurement within the plane are calculated from the time-of-flight and the angle (azimuth and elevation), taking the lidar as the origin. All lidar signal processing was performed using numerical computing software (Matlab^®^ version 7.3, MathWorks Inc., Nastick, MA, USA).

## 3. Results and Discussion

### 3.1. Spray Flux Measurement over the Crop Canopy

**Figure 4:**
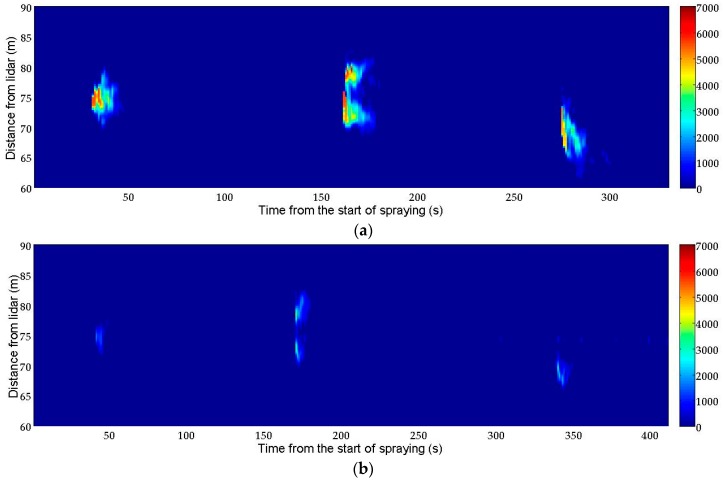
Lidar measurements of the spray flux over the canopy. (**a**) Test T1: standard nozzles; (**b**) Test T2: low-drift nozzles. In both tests, temporal resolution is 1 s and spatial resolution is 2.4 m. In both figures the laser beam goes from bottom to top. Colorbar represents range-corrected backscattered signal (arbitrary units).

Figure 4a shows the RTI plot of the spray plumes corresponding to the test T1 (standard nozzles). Results are consistent with each spraying strategy, shown in Figure 2a. The lidar system was able to distinguish whether the application was made using nozzles on only one or both sides. Likewise, the RTI plot shows the range and temporal evolution of the spray plume, which is a fundamental advantage over passive collectors. Figure 4b presents a lidar measurement corresponding to the test T2 (low-drift nozzles). These results are qualitatively similar to those of test T1, but the backscattered signal intensity (integrated in time and distance for the period and range represented) is 77% lower. This agrees with the fact that by using low-drift nozzles the amount of product that rises above the trees is less.

### 3.2. Comparison Between Spray Drift Measurements Using the Lidar and the ISO 22866 Methodology

**Figure 5:**
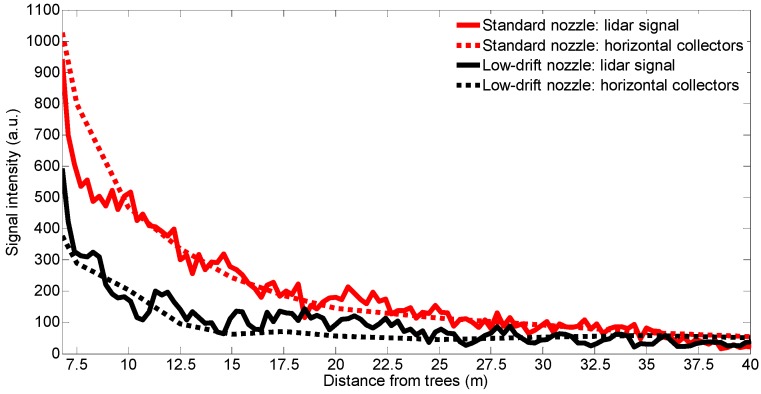
Tests T3 (low-drift nozzles) and T4 (standard nozzles). Time-integrated lidar signal and tracer mass captured by horizontal collectors at each downwind distance from the first tree row (arbitrary units).

Figure 5 compares the time-integrated lidar signal with the measurements carried out by horizontal collectors for a standard nozzle test (T4) and for a low-drift nozzle test (T3). A high coefficient of determination (R^2^ ≈ 0.90) between lidar and horizontal collectors was obtained with standard nozzles. High determination coefficient figures (R^2^ ≈ 0.85) were also obtained with low-drift nozzles, even though the signal is lower in this case, as was expected. With respect to DRP, a value equal to 69% is obtained from depositions on horizontal collectors and a DRP of 57% when the calculation is based on lidar measurements. These results demonstrate that the developed lidar system is a suitable tool for nozzle classification according to their drift potential.

Although the lidar system detected airborne spray drift, no relationship with vertical collector measurements (nylon lines and water sensitive paper sheets) was found. This result is attributed to distortions in lidar signal introduced by the mirror responsible for vertically deflecting the laser beam. This mirror becomes contaminated as the spraying proceeds, altering the deflection and introducing unwanted signal peaks. It is concluded that for airborne drift measurement, RHI (vertical) scans are more appropriate, as shown in Figure 7. RHI scans do not require the use of mirrors or other auxiliary elements.

### 3.3. Lidar Scanning of the Spray Plume over the Crop Canopy

**Figure 6:**
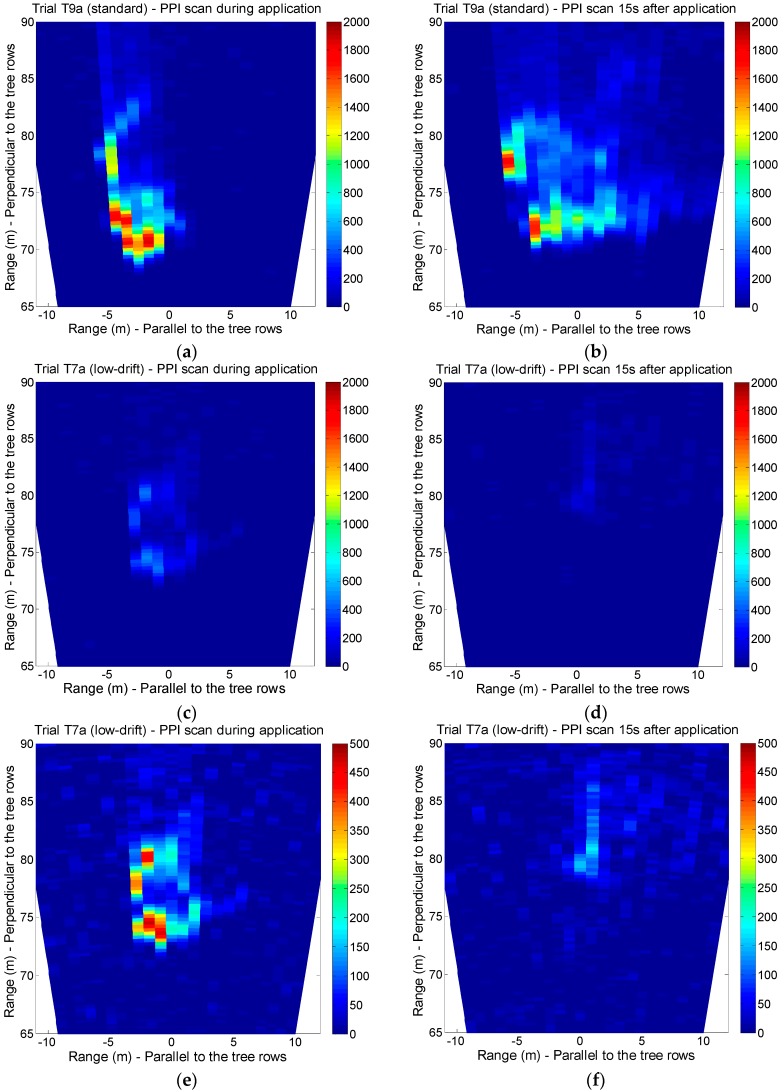
PPI lidar scans of several spray plumes. (**a**) Test T9a (standard nozzles), during the application; (**b**) Test T9a, 15 s after the application; (**c**) Test T7a (low-drift nozzles), during the application; (**d**) Test T7a, 15 s after the application; (**e**) Test T7a, during the application; (**f**) Test T7a, 15 s after the application. Colorbar represents range-corrected backscattered signal (arbitrary units: a.u.). Signal range in (**a**–**d**) is 0–2000 a.u.; in (**e**,**f**) is 0–500 a.u.

Figure 6a,b presents PPI scans corresponding to test T9a (standard nozzles), conducted during the spraying and 15 s after the application, respectively. It can be observed that 15 s after the application, a high fraction of the spray plume remains suspended above the canopy but with a higher degree of dispersion. This phenomenon is repeated in the remaining tests with standard nozzles. Similarly, Figure 6c,d shows PPI scans of trial T7a (low-drift nozzles), represented at the same scale as Figure 6a,b for ease of comparison. An average signal reduction of 75% was computed when comparing low-drift nozzle tests (T7a and T7b) with standard nozzle tests (T9a and T9b). These results are in accordance with the known fact that droplets generated by low-drift nozzles have a lower tendency to disperse over the canopy. Figure 6e,f represent the same measurements as Figure 6c,d, with a narrower signal range that allows to display much more information. It has been demonstrated that the developed lidar system has the ability to detect even the spray plumes generated by low-drift nozzles with a high signal-to-noise ratio.

**Figure 7:**
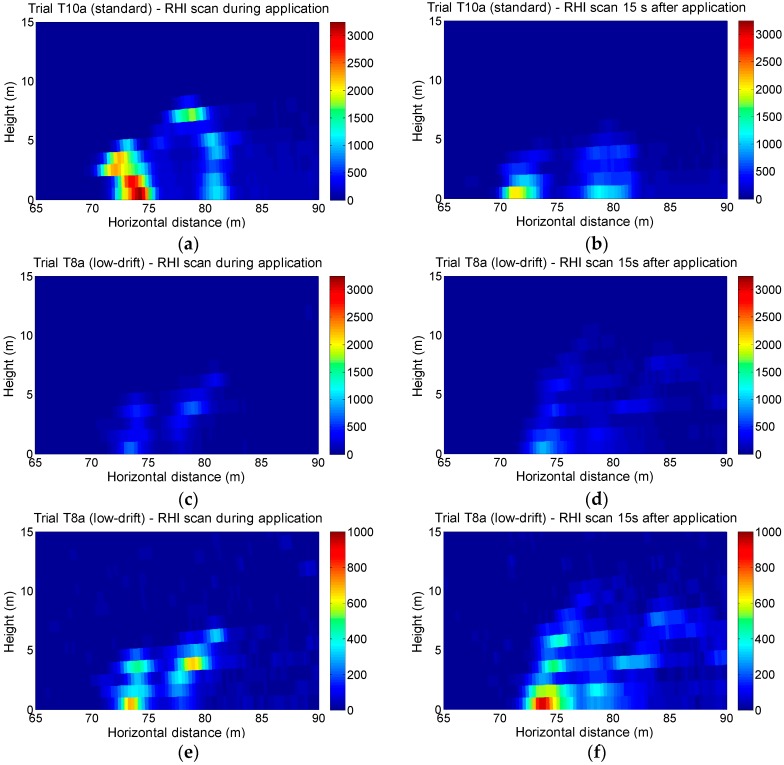
RHI (vertical) lidar scans of several spray plumes. (**a**) Test T10a (standard nozzles), during the application; (**b**) Test T10a, 15 s after the application; (**c**) Test T8a (low-drift nozzles), during the application; (**d**) Test T8a, 15 s after the application; (**e**) Test T8a, during the application; (**f**) Test T8a, 15 s after the application. Colorbar represents range-corrected backscattered signal (arbitrary units: a.u.). Signal range in (**a**–**d**) is 0–3250 a.u.; in (**e**,**f**) is 0–1000 a.u.

Figure 7 presents RHI (vertical) scans corresponding to tests T8a and T10a. In all cases, spray plumes generated by each side of the sprayer are clearly distinguished. In some trials, these plumes reached more than 10 m over the vegetation. In RHI scans, an average signal reduction of 73% was obtained when comparing low-drift nozzle tests (T8a and T8b) with standard nozzle tests (T10a and T10b).

Figure 4, Figure 6 and Figure 7 show that the spray plumes produced by the nozzles on the sprayer side opposite to the lidar system appear more attenuated than the spray plumes produced on the side closer to the lidar system. This is because the plume closer to the lidar system absorbs a fraction of the laser beam energy, and therefore the farthest plume becomes underestimated. A similar problem arises in atmospheric cloud profiling when using, e.g., ground-based ceilometers [38]. However, in the present application, this problem can be solved by independently monitoring the spray drift produced by each side; *i.e.*, with the sprayer working only with the nozzles on the side closer to the lidar system. For sprayers with heterogeneous air distributions, such as the ones equipped with an axial fan, it could be necessary to monitor the spray drift with two subsequent runs, each one with the sprayer using only the nozzles working on the side closer to the lidar system but with opposite directions of motion.

## 4. Conclusions

This work presents the first experimental results obtained with a lidar system specifically designed for spray drift monitoring. In the first experiment, the lidar was operated in staring mode to measure the spray plume over the canopy with high temporal (1 s) and spatial (2.4 m) resolution. The developed sensor has demonstrated its capacity to identify which side of the sprayer was operating as well as the nozzle type used in each test (standard *vs*. low-drift nozzles). In the second experiment, lidar measurements of spray drift were compared with those of the passive collectors, resulting in a strong linear relation (R^2^ > 0.85) between them. These coefficients of determination are similar to those obtained by the authors in a previous study [11], where a commercial ultraviolet lidar system was used. These results are also higher than values presented by Khot *et al.* [29], who compared a 1064 nm lidar system with passive and active collectors (rotorod). The third experiment included 2D scans of the spray flux over the canopy, both in azimuth and elevation. Two-dimensional images of the spray plumes were generated.

Likewise, in the second experiment, a DRP of 57% was computed by comparing lidar measurements in low-drift nozzle tests with those made using standard ones. With regard to the spray plume measurement over the canopy (experiments 1 and 3), signal reductions between 73% and 77% were calculated. Thus, the lidar is presented as an alternative for nozzle and sprayer classification according to their drift potential. Other methods used to evaluate the drift potential, such as droplet size characterization [20,21] or wind tunnel measurements following ISO 22856 standard [39], allow great repeatability since the tests are carried out under controlled conditions. In contrast, field measurements with the lidar system, allow to reproduce more realistic conditions without space limitations.

These results demonstrate that the developed lidar system can replace the currently used passive collectors for the field measurement of spray drift, with significant advantages in terms of performance (high temporal and spatial resolution, scanning capability) and practical application (reduction in the consumption of time and labour, no chemical analysis). However, it must be noted that DRP values presented here are preliminary due to the statistically-limited number of measurement tests. Future work will include further testing to determine DRPs in response to different operation conditions (e.g., nozzle type, nozzle size, operational pressure, *etc.*).

On the other hand, this work has also shown a direct comparison between lidar and passive collector measurements by using a vertical-deflection mirror. This mirror-based configuration is limited by the fact that spray droplets progressively impregnate it. Future tests will include a cleaning mechanism to alleviate this systematic error. Alternatively, this mirror arrangement could be replaced by RHI (vertical) lidar scans sited in place of the mirror.

In addition, this instrument opens the possibility to carry out new studies, such as the assessment of the mass balance in a spray application, the study of factors affecting the persistence of the product in the air, or the analysis of the effect on the spray drift of the spray passes along each alley. These results are encouraging to propose a new lidar-based methodology alternative to the current ISO 22866 standard [1] methodology with passive collectors. This methodology will be possibly based on lidar scanning, since, as shown, it provides more information than staring measurements.

## Figures and Tables

**Table 1:** Experimental field tests.

Test	Date	Start Time (UTC)	Spraying Duration (s)	Lidar Operational Mode			Passive Collectors (ISO 22866 [1])			Sprayer Operational Mode		Nozzles	
				Static with mirror 2 perpendicular directions	Static without mirror: measuring over the trees	Scanning over the trees: scanning plane	Filter paper (horizontal collectors)	Nylon lines (vertical collectors)	Water sensitive papers (vertical collectors)	Moving along crop alleys (# of alleys)	Stationary	Standard Albuz ATR 80 Grey	Low Drift Albuz TVI 8003 Blue
T1	2014-11-13	16:15:31	330	-	X	-	-	-	-	X(2)	-	X	-
T2	2014-11-13	16:38:23	409	-	X	-	-	-	-	X(2)	-	-	X
T3	2014-11-18	13:29:54	802	X	-	-	X	X	X	X(7)	-	-	X
T4	2014-11-18	15:10:51	883	X	-	-	X	X	X	X(7)	-	X	-
T5	2014-11-19	11:50:05	935	X	-	-	X	X	X	X(7)	-	X	-
T6	2014-11-19	14:20:21	823	X	-	-	X	X	X	X(7)	-	-	X
T7a ^1^	2014-11-21	12:19:48	80	-	-	PPI ^2^	-	-	-	-	X	-	X
T7b ^1^	2014-11-21	12:26:18	65	-	-	PPI	-	-	-	-	X	-	X
T8a	2014-11-21	12:43:53	65	-	-	RHI ^2^ (vertical)	-	-	-	-	X	-	X
T8b	2014-11-21	12:46:17	65	-	-	RHI (vertical)	-	-	-	-	X	-	X
T9a	2014-11-21	14:35:33	65	-	-	PPI	-	-	-	-	X	X	-
T9b	2014-11-21	14:42:11	65	-	-	PPI	-	-	-	-	X	X	-
T10a	2014-11-21	14:54:07	65	-	-	RHI (vertical)	-	-	-	-	X	X	-
T10b	2014-11-21	15:00:27	65	-	-	RHI (vertical)	-	-	-	-	X	X	-

^1^ Letters a and b refer to repetitions of the same test. ^2^ PPI and RHI scans are described in Section 2.7 (PPI: Plan position indicator; RHI: Range-height indicator). Lidar: Light detection and ranging.

**Table 2:** Lidar system specifications.

Item	Specification
Wavelength	1534 nm (Erbium glass laser)
Pulse energy	3 mJ
Pulse duration	6 ns
Repetition rate	Single shot-10 Hz (adjustable)
Beam divergence	210 μrad (full angle)
Telescope aperture	80 mm
Detector type	Avalanche photodiode
Sampling rate	500 MS/s
Digitizer resolution	12 bits
Spatial resolution	2.4 m

**Table 3:** Average micrometeorological conditions during the tests.

Test	T (°C) Height = 4 m	T (°C) Height = 7 m	Relative Humidity (%)	Wind Speed (m·s^−1^)	Wind Direction (degrees ^1^)
T1	12.94	12.24	74.27	1.47	−3.42
T2	12.11	11.61	79.89	1.46	−7.74
T3	15.78	15.32	49.98	1.61	−9.43
T4	16.67	16.38	53.51	1.81	−14.21
T5	16.27	15.76	60.98	1.12	−21.83
T6	16.65	15.89	58.00	1.21	−24.07
T7a	15.89	15.30	68.72	1.07	12.04
T7b	16.45	15.91	67.76	1.32	6.52
T8a	17.13	16.73	65.85	1.00	−6.65
T8b	17.32	16.91	64.85	0.93	−17.11
T9a	16.21	15.29	62.92	0.98	18.89
T9b	16.92	16.70	72.97	1.42	−17.77
T10a	16.86	16.62	73.94	1.00	7.23
T10b	16.82	16.52	75.94	1.07	17.23

^1^ Degrees from direction orthogonal to tree rows.

## Abbreviations

The following abbreviations are used in this manuscript:

LIDAR	Light detection and ranging
RTI	Range-time intensity
DRP	Drift reduction potential
PPI	Plan position indicator
RHI	Range-height indicator

## References

[1] International Standard Equipment for Crop Protection. Methods for Field Measurement of Spray Drift. ISO 22866. http://www.iso.org/iso/iso_catalogue/catalogue_tc/catalogue_detail.htm?csnumber=35161.

[2] Makarov V.I., Ankilov A.N., Koutsenogii K.P., Borodulin A.I., Samsonov Y.N. (1996). Efficiency of the inertial wind capture of pesticide aerosols by vegetation species. J. Aerosol Sci..

[3] Unsworth J.B., Wauchope R.D., Klein A.W., Dorne E., Zeeh B., Yeh S.M., Akerblom M., Racke K.D., Rubin B. (1999). Significance of the long range transport of pesticides in the atmosphere. Pure Appl. Chem..

[4] Elliott J.G., Wilson B.J. (1983). The Influence of Weather on the Efficiency and Safety of Pesticide Application: The Drift of Herbicides.

[5] Schulz R. (2004). Field studies on exposure, effects and risk mitigation of aquatic nonpoint-source insecticide pollution: A review. J. Environ. Qual..

[6] Cunha J.P., Chueca P., Garcerá C., Moltó E. (2012). Risk assessment of pesticide spray drift form citrus applications with sir-blast sprayers in Spain. Crop Prot..

[7] Butler-Ellis M.C., Lane A.G., O’Sullivan C.M., Miller P.C.H., Glass C.R. (2010). Bystander exposure to pesticide spray drift: New data for model development and validation. Biosyst. Eng..

[8] Ganzelmeier H., Rautmann D., Spangenberg R., Streloke M., Herrmann M., Wenzelburger H.J., Walter H.F. (1995). Studies on spray drift of plant protection products. Mitt. Biol. Bundesanst. Land. Forstwirtsch. Berl. Dahl..

[9] Felsot A.S., Unsworth J.B., Linders J.B.H., Roberts G., Rautman D., Harris C., Carazo E. (2011). Agrochemical spray drift; assessment and mitigation—A review. J. Eviron. Sci. Health B.

[10] Miller P.C.H., Matthews G.A., Hislop E.C. (1993). Spray drift and its measurement. Application Technology for Crop Protection.

[11] Gregorio E., Rosell-Polo J.R., Sanz R., Rocadenbosch F., Solanelles F., Garcerá C., Chueca P., Arnó J., del Moral I., Masip J. (2014). LIDAR as an alternative to passive collectors to measure pesticide spray drift. Atmos. Environ..

[12] Llorens J., Gallart M., Llop J., Miranda-Fuentes A., Gil E. (2016). Difficulties to apply ISO 22866 requirements for drift measurement. A particular case of traditional olive tree plantations. Asp. Appl. Biol..

[13] May K.R., Clifford R. (1967). The impaction of aerosol particles on cylinders, spheres, ribbons and discs. Ann. Occup. Hyg..

[14] Cooke B.K., Hislop E.C., Matthews G.A., Hislop E.C. (1993). Spray tracing techniques. Application Technology for Crop Protection.

[15] Gil Y., Sinfort C. (2005). Emission of pesticides to the air during sprayer application: A bibliographic review. Atmos. Environ..

[16] Derksen R.C., Ozkan H.E., Fox R.D., Brazee R.D. (1999). Droplet spectra and wind tunnel evaluation of venture and pre-orifice nozzles. Trans. ASAE.

[17] Nuyttens D., Taylor W.A., De Schampheleire M., Verboven P., Dekeyser D. (2009). Influence of nozzle type and size on drift potential by means of different tunnel evaluation methods. Biosyst. Eng..

[18] Balsari P., Marucco P., Tamagnone M. (2007). A test bench for the classification of boom sprayers according to drift risk. Crop Prot..

[19] Gil E., Balsari P., Gallart M., Llorens J., Marucco P., Andersen P.G., Fàbregas X., Llop J. (2014). Determination of drift potential of different flat fan nozzles on a boom sprayer using a test bench. Crop Prot..

[20] Van de Zande J.C., Holterman H.J., Wenneker M. (2008). Nozzle classification for drift reduction in orchard spraying: Identification of drift reduction class threshold nozzles. Agric. Eng. Int. CIGR J..

[21] Planas S., Solanelles F., Torrent X., Camp F., Gregorio E., Rosell J.R. Comparing standardized methods for potential drift assessment. Proceedings of the 12th Workshop on Spray Application Techniques in Fruit Growing.

[22] Hoff R.M., Mickle R.E., Froude F.A. (1989). A rapid acquisition lidar for aerial spray diagnostics. Trans. ASAE.

[23] Mickle R.E. (1994). Utilizing vortex behaviour to minimize drift. J. Eviron. Sci. Health B.

[24] Mickle R.E. (1996). Influence of aircraft vortices on spray cloud behaviour. J. Am. Mosq. Contr..

[25] Stoughton T.E., Miller D.R., Yang Y., Ducharme K.M. (1997). A comparison of spray drift predictions to lidar data. Agric. For. Meteorol..

[26] Miller D.R., Stoughton T.E. (2000). Response of spray drift from aerial applications at forest edge to atmospheric stability. Agr. For. Meteorol..

[27] Miller D.R., Khot L.R., Hiscox A.L., Salyani M., Walker T.W., Farooq M. (2012). Effect of atmospheric conditions on coverage of fogger applications in a desert surface boundary layer. Trans. ASABE.

[28] Hiscox A.L., Miller D.R., Nappo C.J., Ross J. (2006). Dispersion of fine spray from aerial applications in stable atmospheric conditions. Trans. ASABE.

[29] Khot L.R., Miller D.R., Hiscox A.L., Salyani M., Walker T.W., Farooq M. (2011). Extrapolation of droplet catch measurements in aerosol application treatments. Atomization Spray.

[30] Gil E., Llorens J., Llop J., Fàbregas X., Gallart M. (2013). Use of a terrestrial lidar sensor for drift detection in vineyard spraying. Sensors.

[31] Gregorio E., Rocadenbosch F., Sanz R., Rosell-Polo J.R. (2015). Eye-safe lidar system for pesticide spray drift measurement. Sensors.

[32] Safety of Laser Products—Part 1: Equipment Classification and Requirements. IEC 60825-1. https://webstore.iec.ch/publication/3587.

[33] Measures R.M. (1992). Laser Remote Sensing: Fundamentals and Applications.

[34] Collis R.T.H., Russell P.B., Hinkley E.D. (1976). Lidar measurement of particles and gases by elastic backscattering and differential absorption. Laser Monitoring of the Atmosphere.

[35] Gregorio E., Torrent X., Solanelles F., Sanz R., Rocadenbsch F., Masip J., Ribes-Dasi M., Planas S., Rosell-Polo J.R. First measurements with a lidar systems specifically designed for spray drift monitoring. Proceedings of the 13th Workshop on Spray Application Techniques in Fruit Growing.

[36] Nuyttens D., De Schampheleire M., Baetens B., Sonck B. (2007). The influence of operator controlled variables on spray drift from field crop sprayers. Trans. ASABE.

[37] Spuler S.M., Mayor S.D. (2005). Scanning eye-safe elastic backscatter lidar at 1.54 μm. J. Atmos. Ocean. Technol..

[38] Gregorio E., Rocadenbosch F., Tiana-Alsina J., Comerón A., Sanz R., Rosell J.R. (2012). Parameter design of a biaxial lidar ceilometer. J. Appl. Remote Sens..

[39] International Standard Equipment for Crop Protection. Methods for the laboratory measurement of spray drift—Wind tunnels. ISO 22856. http://www.iso.org/iso/iso_catalogue/catalogue_tc/catalogue_detail.htm?csnumber=41187.

